# ZIF-8-Based Surface Plasmon Resonance and Fabry–Pérot Sensors for Volatile Organic Compounds

**DOI:** 10.3390/s24134381

**Published:** 2024-07-05

**Authors:** Anna Estany-Macià, Ignasi Fort-Grandas, Nirav Joshi, Winnie E. Svendsen, Maria Dimaki, Albert Romano-Rodríguez, Mauricio Moreno-Sereno

**Affiliations:** 1 Department of Electronics and Biomedical Engineering, Universitat de Barcelona, 08028 Barcelona, Spain; ignfortgra_9@ub.edu (I.F.-G.); niravjoshi@ub.edu (N.J.); 2 Institute of Nanoscience and Nanotechnology (IN2UB), Universitat de Barcelona, 08028 Barcelona, Spain; 3 Department of Inorganic and Organic Chemistry, Universitat de Barcelona, 08028 Barcelona, Spain; 4 Group NABIS, Department of Biotechnology and Biomedicine, Technical University of Denmark (DTU), 2800 Kongens Lyngby, Denmark; wisv@dtu.dk (W.E.S.); madi@dtu.dk (M.D.)

**Keywords:** gas sensor, MOFs, ZIF-8, optical detection, Fabry–Pérot interferometer, surface plasmon resonance, VOCs, ethanol

## Abstract

This work explores the use of ZIF-8, a metal–organic framework (MOF) material, for its use in the optical detection of volatile organic compounds (VOCs) in Fabry–Pérot and surface plasmon resonance (SPR)-based sensors. The experiments have been carried out with ethanol (EtOH) and show response times as low as 30 s under VOC-saturated atmospheres, and the estimated limit of detection is below 4000 ppm for both sensor types. The selectivity towards other VOCs is relatively poor, although the dynamics of adsorption/desorption differ for each VOC and could be used for selectivity purposes. Furthermore, the hydrophobicity of ZIF-8 has been confirmed and the fabricated sensors are insensitive to this compound, which is a very attractive result for its practical use in gas sensing devices.

## 1. Introduction

Volatile organic compounds (VOCs) are chemical compounds that contain at least one carbon atom and a hydrogen atom in their molecular structure, and their relatively low boiling point allows evaporation or sublimation into air. Even though the most significant sources of VOCs can be found in indoor environments, e.g., building materials, paints, household products, tobacco smoke, cooking, clothing, or cosmetics, they can also be found in a wide range of outdoor sources, such as industrial processes or vehicle emissions [1,2].

Since exposure to VOCs is largely through inhalation, the quality of the air is directly related to our health, and even though most of them do not pose a serious health risk, long-term exposure can lead to severe health problems. In fact, VOCs have been linked to acute and long-term health issues which include irritation, carcinogenic and mutagenic effects such as acute myeloid leukemia, and respiratory effects like reduced pulmonary function or asthma [2,3]. 

Environmentally, VOCs contribute to global warming by the absorption of infrared radiation. In addition, they can react with nitrogen oxides, leading to the formation of ground-level ozone, which is a strong greenhouse gas, and can also oxidize to NO_2_, resulting in increased levels of NO in air. The oxidizing and corrosive properties of VOCs, combined with the formation of ozone, can attack materials and cause their accelerated degradation, i.e., protective layers in buildings. Additionally, larger-size particulate matter formation can occur due to the VOCs’ role as an organic precursor gas for particle growth [4].

Therefore, the analysis of VOCs is key to monitor air quality and identify sources of pollution. Currently, gas chromatography coupled with mass spectroscopy (GC-MS) is the established analytical method, which provides qualitative and quantitative information while being an expensive, bulky, complex-to-operate and not portable technique. While other commercially available methods are smaller in size and sensitive to a range of VOCs with a short response time, e.g., photoionization detectors (PIDs) and metal-oxide sensors, their main limitation is cross-sensitivity towards multiple gases, which severely affects their response, and no qualitative information on the nature of the VOC can be obtained. To overcome these challenges, a wide range of gas sensors are currently being developed, such as surface acoustic wave sensors [5,6,7,8], infrared spectroscopy [9,10,11], quartz microbalance sensors [12,13,14,15], electrochemical sensors [16,17,18,19,20,21], colorimetric sensors [22,23], or fluorescent sensors [24,25,26]. Meanwhile, other sensing platforms are emerging to overcome the lack of sensitivity by combining multiple sensors that can identify and quantify individual components in gas mixtures, and via pattern recognition systems, the data are then analyzed and correlated to results obtained from other techniques [27]. 

The difficulty of detecting VOCs lies in their low partial pressure and low surface tension [28]; hence, one pathway to enhance the response of gas sensing devices consists of the functionalization of the sensor’s surface with a sensitive coating, e.g., metal oxides, polymers, zeolites or metal–organic frameworks (MOFs) [20], which not only can increase the concentration of gas molecules on the surface to improve sensitivity (preconcentration) but can also tailor the selectivity via chemical affinity [29].

MOFs are porous materials with a crystalline structure formed by metal nodes (metal ions) connected through organic ligands. The appeal of MOFs for gas sensing applications relies on their intrinsic porosity, i.e., their high surface-to-volume ratio, which favors diffusion, adsorption, and interaction with analytes, and the tunable pore size, which can naturally block larger molecules outside their pores (filtering) and therefore contributes to increased selectivity [30,31]. Plus, in contrast to the high operating temperatures required for other materials used as sensitive coatings for gas detection, e.g., metal oxides [18,19,20], MOFs can be used for gas sensing applications at room temperature. In fact, several MOF-based sensors employing different sensing mechanisms have been developed in the last 20 years. Even though the insulating character of MOFs hinders their use in electrochemical sensors [32], other approaches that are based on luminescent or colorimetric sensing based on intermolecular interactions, redox-active ligands, or charge transfer between MOFs and adsorbed VOC molecules have been reported [33,34,35]. Other techniques rely on monitoring the bulk refractive index (RI) change of the MOFs with the absorption of guest molecules. In this manner, micro resonators [36], photonic structures using engineered colloidal MOF films [37,38,39,40,41], or sensors with surface-enhanced Raman scattering [42,43] have been developed for gas sensing applications. Specifically, Fabry–Pérot (FP) interferometry was used in the first proof of concept of MOFs for VOC sensing by Hupp et al. in 2010 [44], in which the MOF film served as the FP cavity and sensitive medium, which absorbed the gas molecules and, thus, modified the FP interference fringes by red-shifting them due to an increase in the MOF RI [45,46,47,48]. Another commonly used RI modulation sensing mechanism in gas detection is surface plasmon resonance (SPR)-based sensors, in which light triggers and confines plasmon oscillation at the interface of a metal and the MOF, and changes in RI in the MOF film due to absorbed analytes red shift the resonance wavelength of plasmons, allowing ppm detection of VOCs and other gases, e.g., CO_2_ [28,31]. Even though there are several configurations of SPR devices, optical-fiber-based SPR sensors are the most used design for gas sensing due to their low attenuation, high sensitivity, and easy integrability into an optical transmission system [28,49,50,51], whereas limited research has been carried out employing grating coupler-based or prism coupler-based SPR configurations for gas sensing applications [52,53,54]. 

In this work, Zeolitic Imidazolate Framework-8 (ZIF-8), which is an interesting MOF for gas sensing applications due to its hydrophobic properties, is used to detect mainly ethanol (EtOH) vapors at room temperature. ZIF-8 films have been grown on two different substrates and a comparison has been drawn between two RI modulation sensing techniques: FP interferometry, by using silicon substrates as FP mirrors and monitoring the interference peaks, and SPR, by working with substrates containing diffraction gratings (DGs) and tracking the plasmon resonance wavelength under different gas mixtures.

## 2. Materials and Methods

### 2.1. Sensing Substrates

The substrates for RI modulation via SPR wavelength shift contained 4 DGs. The fabrication process consisted of a low-pressure chemical vapor deposition (LPCVD) of 100 nm of Si_3_N_4_ on top of an oxidized silicon wafer (900 nm SiO_2_). Next, a 180 nm thick CSAR AR P6200-09 photoresist was spun on top, followed by thermal evaporation of a 20 nm thick aluminum (Al) layer. Then, patterning via electron beam lithography (Jeol JBX-9500FS, Jeol Ltd., Tokyo, Japan) was performed, the Al layer was removed in a TMAH solution, and the photoresist was developed using Developer AR 600-546 (Allresist GmbH, Strausberg, Germany). The upper 30 nm of the Si_3_N_4_ was then etched inside an RIE system, using CHF_3_ chemistry. Finally, the photoresist was stripped off and a metal stack of Ti (3 nm)/Au (40 nm) was deposited via e-beam evaporation. The pattern consisted of two different grating periods, Λ = 400 nm and Λ = 500 nm, in a square area of 0.5 mm × 0.5 mm, which was repeated twice per chip. The Si_3_N_4_–metal stack interface works as an SPR device (SPR-G samples).

The substrates for RI modulation via shift of FP interferences were silicon (Si) substrates, on top of which ZIF-8 films were deposited (FP samples).

### 2.2. ZIF-8 Growth on Substrates

Prior to any synthesis, the substrates were cleaned with acetone and isopropanol in 10 min ultrasonic baths and dried under nitrogen (N_2_) flow. Cleaned substrates were used immediately.

Even though several approaches to growing ZIF-8 have been reported, some of which include further treatment and functionalization of the surface [55,56,57], the method reported by Hupp et al. [44] was used to synthesize ZIF-8. In this way, each ZIF-8 growth cycle was performed by immersing the cleaned substrates in a solution of 2-methylimidazole (50 mM) and Zn(NO_3_)_2_·6H_2_O (25 mM) in a 1:1 volume mixture for 30 min at room temperature. The samples were then rinsed with pure methanol and dried with N_2_ flow. Thicker films were obtained by repeating this same process using freshly prepared solutions in each new growth run.

**Figure 1:**
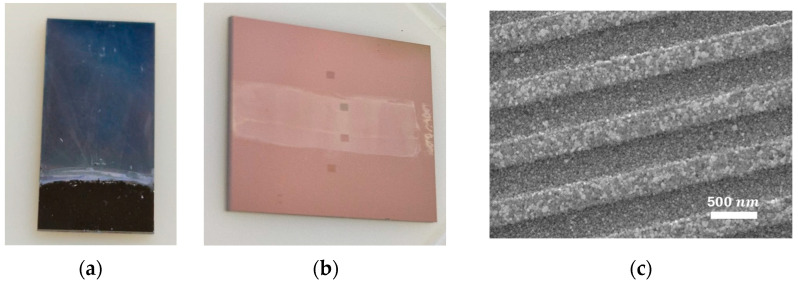

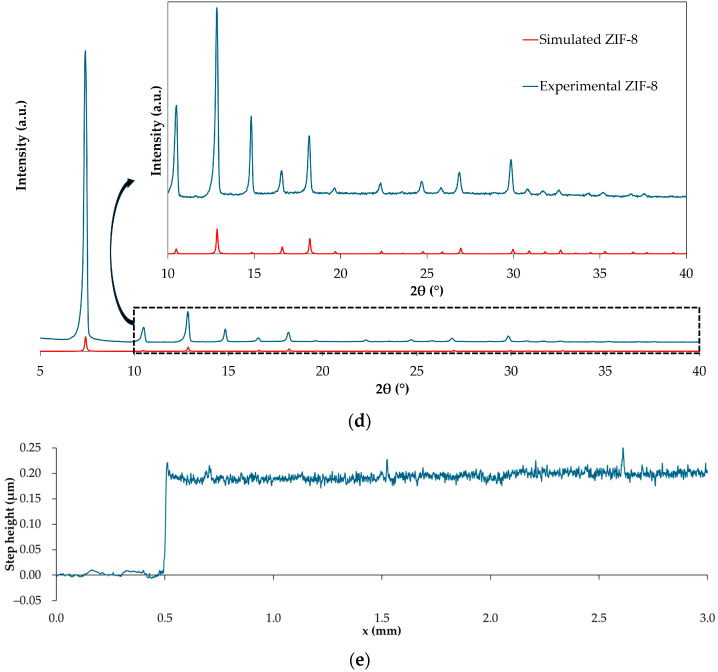
(**a**) The 1 × 1.5 cm^2^ Si substrate covered with around 1550 nm ZIF-8 film; the lower part is the reference for profilometry, etched in nitric acid; (**b**) 1.5 × 1.5 cm^2^ chip containing 4 DGs: two bottom gratings with period Λ = 400 nm and two top ones, with Λ = 500 nm. The two central DGs are covered with around 200 nm ZIF-8 film (lighter region in the image); (**c**) SEM image of uncoated Λ = 400 nm DG. (**d**) X-ray diffractogram of 1550 nm ZIF-8 film deposited on silicon substrate matched with the simulated diffractogram (JCPDS: 00-062-1030). (**e**) Step profile for a sample subjected to 2 ZIF-8 growth runs, giving rise to a 200 nm film.

As optical measurements usually require reference spectra, a ZIF-8-free area in the sample needs to be available. For this, part of the substrates was further immersed in a diluted nitric acid mixture (68% nitric acid/H_2_O at 1:1000 (*v*/*v*)) for 5 s to remove the already deposited ZIF-8 [45]. The resulting samples can be seen in Figure 1a,b.

The crystallinity of the film was examined by X-ray diffraction (XRD) using a PANalytical X’Pert PRO MPD alpha 1 (Malvern, Almelo, The Netherlands) powder diffractometer in Bragg–Brentano θ/2θ geometry, Ni-filtered Cu Kα radiation (λ = 1.5418 Å, 45 kV and 40 mA), a diffracted-beam 0.04-radians Soller-slit collimator, and a PIXcel Detector (active length = 3.347°). The samples were scanned over a 2θ range of 5° to 40°, with a 2θ step size of 0.026° and a step time of 250 s. The XRD patterns (Figure 1d) establish that the position and intensity of the peaks are in good agreement with the modeled XRD pattern for ZIF-8.

To characterize the thickness of the deposited ZIF-8 film, a stylus profilometer (Dektak XT, Bruker, Billerica, MA, USA) was used to measure a 2 mm long line perpendicular to the ZIF-8-free/ZIF-8 border. The obtained results indicate that the layer’s growth is in agreement with the reported deposition rate of 100 nm per 30-minute cycle in the literature [44,47,49,58].

### 2.3. Experimental Set-Up and Signal Processing

**Figure 2:**
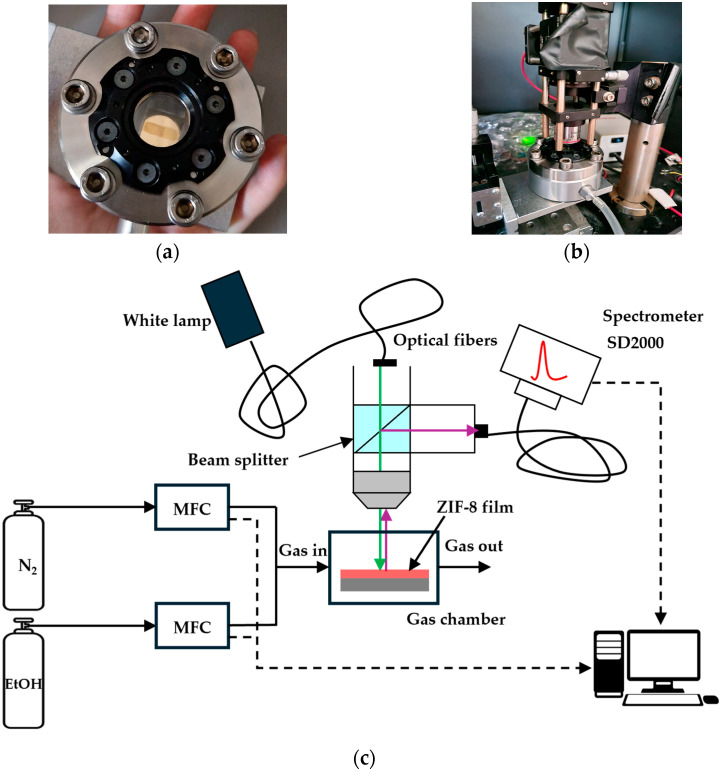
(**a**) Chamber for gas testing containing an SPR-G sensor with the central DG covered with ZIF-8 (shown in Figure 1b). (**b**) Detail of the microscope objective on the top of gas chamber for performing reflectance measurements. (**c**) Sketch of optical set-up employed for the reflectance measurements.

All gas sensing measurements were conducted via spectral interrogation on the fabricated samples at room temperature. These were placed in a holder inside a home-built aluminum chamber with a quartz window, which was attached to an aluminum platform controlled by two motors that allowed moving the sample along the x-y axes, so that fine light beam positioning on certain spots, especially on the DG in the SPR-G samples, could be achieved. The gas chamber was also connected to two 200 sccm Bronkhorst mass flow controllers (MFCs), connected to pure N_2_ and 10,000 ppm EtOH in N_2_ bottles. White light (Ocean Optics LS-1, Ocean Insight, Orlando, FL, USA), which was allowed to thermally stabilize for 1 h prior to any experiment, was focused on an optical fiber, collimated, and further divided with a beam-splitter and TM polarized, which is required for SPR-G samples. One of the beams was focused on the sample’s surface through the quartz window of the gas measuring chamber using a 4× objective. An Ocean Optics SD2000 spectrometer (Ocean Insight, Orlando, FL, USA) acquired the reflectivity spectra (Figure 2).

The alcohol vapor testing experiments were carried out by first purging the chamber, using pure N_2_ (200 sccm), followed by acquiring the reference spectrum in a ZIF-8-free area (bare substrate). The measurement was continued by capturing the response and recovery of the ZIF-8-covered region when VOC pulses were introduced in two different approaches:

1. Approach I: A total of 1 μL of different EtOH/water solutions was manually added into the chamber with a micropipette without contacting the sample and was naturally allowed to evaporate. For this, the chamber was rapidly closed after the introduction of the VOC.
2. Approach II: N_2_ and EtOH gas flows were controlled with the MFCs to introduce different EtOH concentration pulses into the chamber by adjusting the flow of the individual MFCs. The flow rate was always maintained at 200 sccm.

The cleaning of the chamber in both approaches is achieved by flowing 200 sccm of N_2_. The measuring system was controlled via a home-developed MATLAB software, which processed the acquired reflected spectra in real time and normalized them to the reference spectrum previously acquired in a ZIF-8-free area in a N_2_ atmosphere. Another home-developed LabVIEW software controlled the MFCs and regulated the gas pulses entering the chamber. 

The spectra were then further processed by averaging the raw signal obtained by the spectrometer and a polynomic approximation was employed to precisely find the FP interference minimums (in FP samples) and SPR wavelength (in SPR-G samples) in a desired spectrum range.

## 3. Results and Discussion

### 3.1. FP Samples

**Figure 3:**
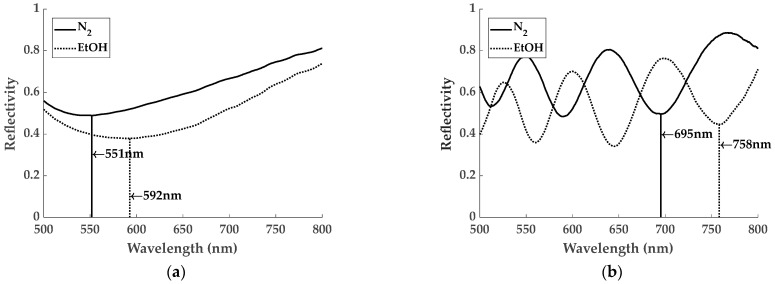

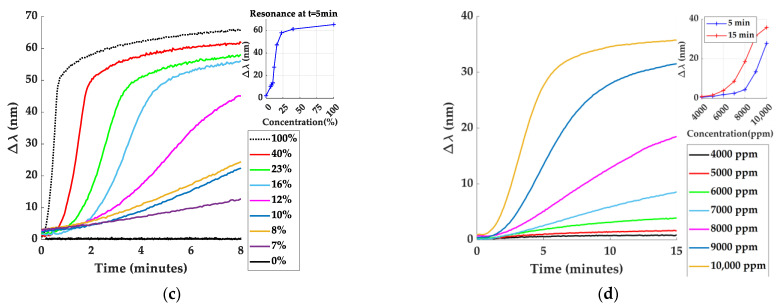
Reflectivity spectra under N_2_ and saturated EtOH atmospheres for samples with (**a**) 670 nm and (**b**) 1550 nm thick ZIF-8 film. Spectra under saturated EtOH atmosphere red-shift by 45 nm and 63 nm in relation to the measurements under pure N_2_, respectively. Response of the 1550 nm thick ZIF-8 covered FP sample towards experiments of (**c**) approach I and (**d**) approach II. The full experiments can be seen in Figure S1.1 and Figure S1.2, respectively.

The ZIF-8 films can be considered as a Fabry–Perot cavity structure, in which light reflections will be caused by changes in RI at the substrate/film and film/air interfaces. Therefore, variations in the ZIF-8 film thickness will lead to different optical paths of the light, which will strongly affect the interferences shown in the reflectivity spectra [40]. From the point of view of gas sensing, gas absorption depends heavily on the number of pores. Therefore, considering a constant pore density, thicker films would be preferred, contributing to larger RI changes and, consequently, larger optical shifts would be obtained. This effect is illustrated in Figure 3a,b, where the reflectivity spectra of 670 and 1550 nm thick ZIF-8 films red shift by 41 and 63 nm, respectively, between the measurements in N_2_ and in the saturated EtOH atmosphere. In addition, the interference pattern for the thicker sample facilitates the localization of a minimum, whose shift can be easily monitored. For this reason, all gas sensing experiments were carried out with the 1550 nm ZIF-8 film, which was the thickest that was fabricated. Even thicker films can negatively impact the dynamics of the analyte transport within the ZIF-8 structure, giving rise to longer response times of the sensors. Therefore, a compromise must be made between thickness and time response to obtain optimal sensor performance.

Taking into consideration the fact that the minimum indicated in Figure 3b is the one that red-shifts the most under VOC exposure (Figure S1.1), it has therefore been taken as the response of the sample. The results obtained for EtOH concentrations ranging from 0 to 100% and from 4000 to 10,000 ppm for approaches I and II, respectively, are shown in Figure 3c,d. As shown, responses up to 66 nm have been obtained for 100% EtOH in approach I, which has been estimated to correspond to 30,000 ppm. These results are comparable to those reported in the literature [44,49], while the responses are larger for those obtained by other optical techniques [40,51,59]. In contrast, approach II led to smaller responses of the sensor signal, producing a red-shift of 1.52 nm at 4000 ppm of EtOH, which is the lowest concentration tested so far (an estimation of the LoD has also been carried out; see Figure S2.1). 

Preliminary simulations have also been made to adjust the experimental curves of the 1550 nm ZIF-8 film to theoretical Fabry–Pérot interferometry curves [48] (Figure S3.1), and the RI values of 1.24 and 1.35 have been obtained for ZIF-8 under N_2_ and saturated EtOH, which is in accordance with other reported ZIF-8 RIs [36,47,60,61,62,63]. 

In addition, while the response time of the sensor in approach I can be as fast as 30 s under 100% EtOH, the longer response time of the sensor in approach II is due to the time needed to reach stationary conditions in the gas chamber with the lower EtOH concentrations of the bottle (maximum 10,000 ppm).

### 3.2. SPR-G Samples

**Figure 4:**
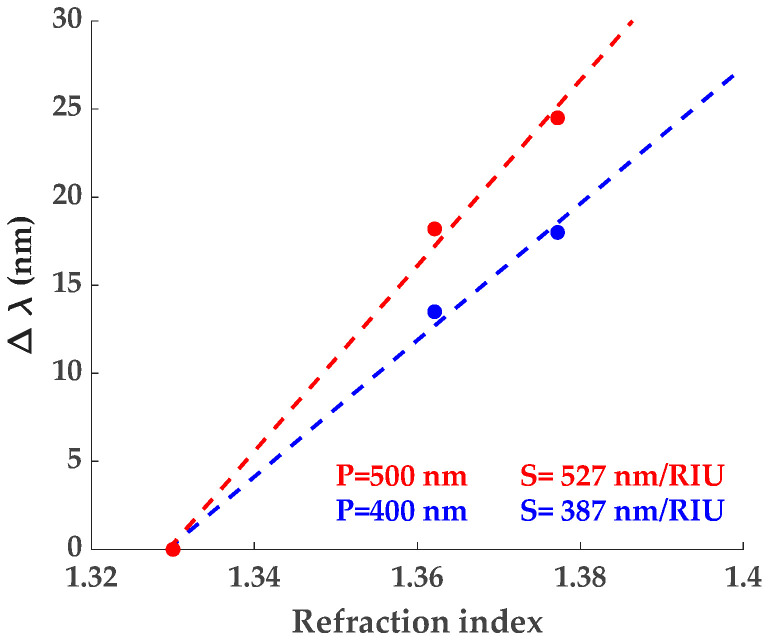
RDW red-shift of Λ = 400 nm and Λ = 500 nm DG for different liquids—water (*n* = 1.33), 25% glycerol (*n* = 1.362, [64]), and isopropanol (*n* = 1.377)—and their bulk sensitivity (S). Water has been taken as reference. RIs of water and isopropanol have been extracted from [65].

In SPR-based sensors, plasmon oscillations confined at the metal/medium interface (MOF in our case) create an evanescent wave whose amplitude decreases exponentially with increasing distance from the interface into the medium. This establishes a maximum depth at which plasmons are sensitive to optical variations in the medium, i.e., resonance dip wavelength (RDW) red-shifts with increasing RI. For calibration, different liquids were used as external medium to obtain the bulk sensitivity of the bare SPR-G substrates (without ZIF-8), which was determined to be around 387 nm/RIU and 527 nm/RIU (Refractive Index Units) for DG of Λ = 400 nm and Λ = 500 nm, respectively (Figure 4).

**Figure 5:**
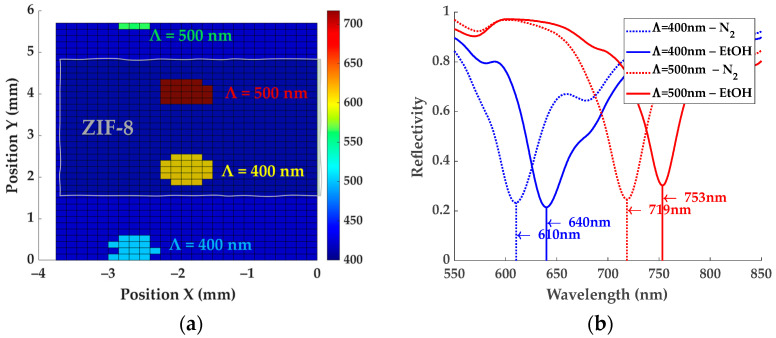
(**a**) SPR-G surface scan with the 4 localized DGs and their RDWs for a ZIF-8 thickness of 300 nm. (**b**) Reflectivity spectra showing the red-shift of the previous RDW under an EtOH saturated atmosphere for both grating periods.

After covering the central DG with a ZIF-8 film and leaving the outer two uncovered for reference, the red-shift caused by the higher RI of the MOF in relation to air can be seen in Figure 5a, which is a scan of a 4 × 6 mm^2^ area of the SPR-G surface in which the four DGs appear in a color representative of their respective RDW: Λ = 400 nm shifts from 500.5 nm to 610.1 nm and Λ = 500 nm from 563.2 nm to 718.8 nm between in-air and 300 nm ZIF-8-covered DGs, respectively. The blue background (λ = 400 nm) represents the flat surface of the substrate, in which no DGs are present and, thus, no resonance is observed.

Gas sensing experiments, which are conducted on the ZIF-8-covered DG, will further redshift the RDW due to the RI change of ZIF-8 during the adsorption/desorption of analyte molecules (Figure 5b). This red-shift will be independent of the ZIF-8 thickness if the films are thicker than the penetration depth of the evanescent wave in the MOF, since no optical changes farther than that will be measured by the SPR-G sensor (no sensitivity).

**Figure 6:**
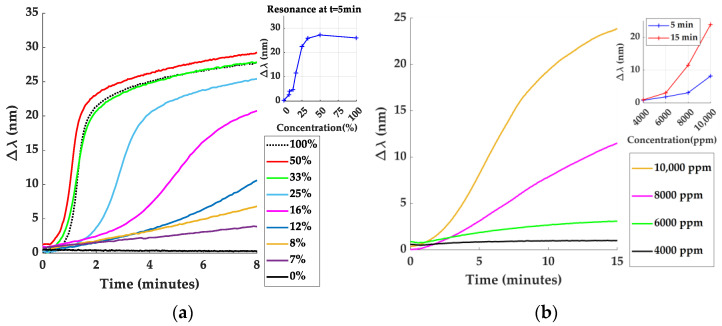
Response of 300 nm ZIF-8-covered SPR-G sample towards (**a**) experiment approach I in Λ = 400 nm DG and (**b**) experiment approach II in Λ = 500 nm DG. For comparison, the complete results of approach I in Λ = 400 nm DG can be seen in Figure S4.1 and in Λ = 500 nm DG in Figure S4.2. Full experiment approach II can be seen in Figure S4.3.

Figure 6 shows the results obtained for EtOH concentrations ranging from 0 to 100% and from 4000 to 10,000 ppm for experiment approaches I and II and Λ = 400 nm and Λ = 500 nm DG, respectively. The results prove dynamics and response times similar to those obtained in FP samples. It is noteworthy that, contrarily to the expected behavior expected given the higher sensitivity of the gratings with Λ = 500 nm DG in front of those with Λ = 400 nm, similar responses have been obtained for both DGs in gas sensing experiments, with the red-shift for gratings with Λ = 500 nm being only slightly larger (Figure S4.2). This fact would allow us to fine tune the working RDW by changing the period of the DG with the miniaturization of the set-up with LEDs and a photodetector (instead of white light and a spectrometer) in mind, without largely affecting the sensitivity towards VOCs.

An additional benefit of this result is that due to the fact that the SPR-G samples only sense the changes in the first few nm from the surface of the ZIF-8 film, in which the evanescent wave penetrates, the film thickness can be reduced to promote good kinetics of adsorption/desorption of the analyte in the ZIF-8 material without impacting the sensitivity of the sensors.

### 3.3. Selectivity, Reversibility, and Reusability of the Samples

**Figure 7:**
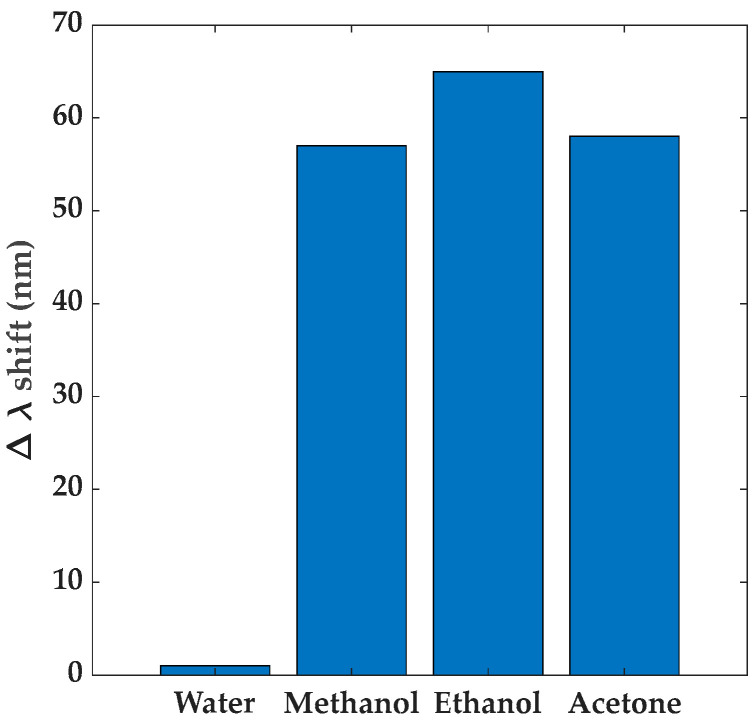
Response of FP sample with 1550 nm ZIF-8 film towards water and different VOCs.

Experiments performed to test the sensor’s response towards other saturated VOC atmospheres were conducted with approach I and FP samples by using other VOCs, like methanol (kinetic diameter, d = 3.54 Å) and acetone (kinetic diameter, d = 6.16 Å), and both induced red-shifts similar to those of EtOH (kinetic diameter, d = 4.18 Å), indicating the poor selectivity of ZIF-8 (Figure 7). However, differences in the dynamic adsorption of the molecules were observed: the response to acetone took around 12 min to reach saturation and 2 h was required to fully recover the initial spectrum of the sample, contrasting with the fast response (around 2 min) and recovery (less than 30 s) of the smaller molecules. This behavior can be attributed to the size of the VOC molecules, as larger molecules will be partly blocked at the ZIF-8 pores due to their larger dimensions [40].

Consistent with the reported hydrophobicity of ZIF-8 [40,44,59], the samples were insensitive to water vapor and, consequently, the red-shift of the reflectivity spectra can only be attributed to the VOC vapors inside the chamber.

The reversibility and reusability of the samples was also observed during the experiments, since alternating pulses of N_2_ and VOC-containing N_2_ did not significantly affect the initial baseline. Therefore, ZIF-8 is advantageous in opposition to other MOF structures which often need heating or molecule evacuating under vacuum to regenerate the samples [39,41]. Long-term measurements for studying the stability of the sensors are ongoing.

## 4. Conclusions and Future Work

In this study, a comparison has been drawn between FP-based sensors and SPR-based sensors for VOC detection. Even though the lowest tested EtOH concentration is 4000 ppm with the current monitoring system and displays time responses that range from a few seconds (with high concentrations) to several minutes (with low concentrations), a difference can be observed between the response of FP and SPR-G samples. In FP samples, the thicker the ZIF-8 film, the larger the number of pores that contribute to RI change, and therefore, a larger optical shift is obtained. Even though this can have a negative effect on the dynamics of the analyte transport through the ZIF-8 layer, thicker films are preferred since they are not only more sensitive but also more precise. In contrast, even though SPR-G samples are more complex and expensive to fabricate, they do not require thick MOF films to enhance their sensitivity: a 300 nm ZIF-8 film on SPR-G samples red-shifts around 32 nm, for which a 670 nm thick film in FP samples is required. Hence, SPR-G samples produce larger optical shifts even with thin ZIF-8 thickness, so that film growth time can be reduced and kinetics is not compromised. Even though the sensitivity of the sensors is comparable to that of other similar sensors [44] and higher than other reported approaches [40,49,51], it is still far from that of ppm and/or ppb detection using fiber-optic-based SPR sensors. However, the planar geometry of SPR-G sensors allows for multiplexed sensing on an array of DGs with different surface functionalization and film thicknesses, which is not possible in fiber-optic-based sensors.

The first results of the selectivity of ZIF-8 towards different VOCs show poor results, and only the dynamics of adsorption of the gas molecules appear to differ depending on the VOC, which could be a method to further distinguish between different adsorbed VOCs. Furthermore, the hydrophobicity of the MOF remains a main attraction for its use in gas sensing devices, since humidity dramatically impacts the performance of many gas sensors.

Future work will focus, on the one hand, on the optimization of the fabrication process and the improvement of the LOD and, on the other, on the miniaturization of the sensing system with SPR-G samples via LEDs and photodiodes to obtain portable and smaller gas sensing systems.

## Data Availability

The original contributions presented in the study are included in the article, further inquiries can be directed to the corresponding authors.

## Supplementary Materials

The following supporting information can be downloaded at https://www.mdpi.com/article/10.3390/s24134381/s1: Figures S1.1–S1.2: Gas sensing experiments in FP samples; Figure S2.1: Sigmoidal fitting of experimental spectral shifts under EtOH pulses for LoD estimation; Figure S3.1: Fabry–Pérot interferometry to obtain RI of ZIF-8 film; Figures S4.1–S4.3: Gas sensing experiments in SPR-G samples.
