# Supplementary material for: ZIF-8-Based Surface Plasmon Resonance and Fabry–Pérot Sensors for Volatile Organic Compounds
Source: Sensors (Basel). 2024 Jul 5;24(13):4381. doi: 10.3390/s24134381 (PMC11244607; doi:10.3390/s24134381)
Supplement: Supplementary file 1 [file sensors-24-04381-s001.zip › sensors-3036979-supplementary.pdf]

Supplementary materials

ZIF-8-based Surface Plasmon Resonance and Fabry–Pérot sensors for volatile organic compounds

Anna Estany-Macià [1, 2,*], Ignasi Fort-Grandas [1,2,3], Nirav Joshi [1,†], Winnie E. Svendsen [4], Maria Dimaki [4], Albert Romano-Rodríguez [1,2,*] and Mauricio Moreno-Sereno [1,2,*]

1 Department of Electronics and Biomedical Engineering, Universitat de Barcelona, 08028 Barcelona, Spain; ignfortgra_9@ub.edu (I.F.-G.), niravjoshi@ub.edu (N.J.)
2 Institute of Nanoscience and Nanotechnology (IN2UB), Universitat de Barcelona, 08028 Barcelona, Spain;
3 Department of Inorganic and Organic Chemistry, Universitat de Barcelona, 08028 Barcelona, Spain;
4 Group NABIS, Department of Biotechnology and Biomedicine, Technical University of Denmark (DTU), 2800 Kongens Lyngby, Denmark; wisv@dtu.dk (W.S.), madi@dtu.dk (M.D.)
† Present address: Luxembourg Institute of Science and Technology, 4362 Esch-sur-Alzette, Luxembourg;
* Correspondence: anna_estany@ub.edu (A.E.-M), albert.romano@ub.edu (A.R.-R), mauricio.moreno@ub.edu (M.M.-S)

S1. Gas sensing measurements in FP samples

Gas sensing measurements have been performed on the FP sample covered with 1550 nm of ZIF-8 via two different approaches. In approach I (Figure S1. 1), 1 µl of different EtOH/water solutions was manually added into the chamber with a micropipette without contacting the sample and was naturally allowed to evaporate. N_2 flow was then used to clean the chamber in between concentrations. In approach II (Figure S1. 2), different EtOH concentrations were introduced in gas form into the chamber (concentrations were adjusted by controlling N_2 and EtOH flows from the bottles and maintaining the flow rate at 200 sccm). N_2 flow was also used to clean the chamber in between pulses of EtOH.

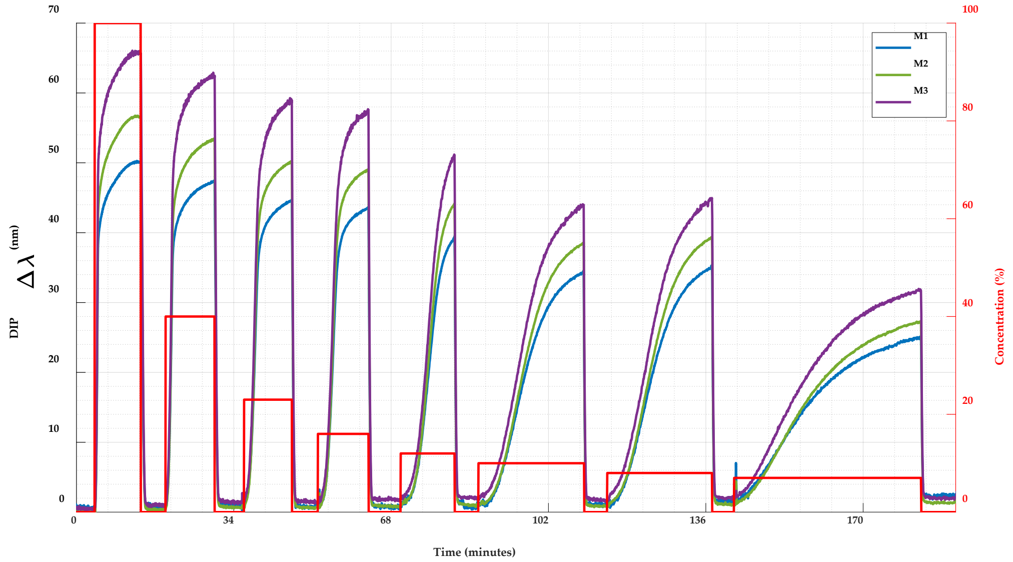

Figure S1.1 Gas sensing approach I. Red shift of the 3 interference minimums of the FP sensor with a 1550 nm ZIF-8 film (Figures 3b and 3c) towards EtOH pulses of 100%, 40%, 23%, 16%, 12%, 10%, 8%, and 7%, respectively. M1, M2, and M3 are the minimums at λ = 512 nm, λ = 589 nm, and λ = 695 nm in N_2, respectively. M3 has been taken as the response of the sensor.

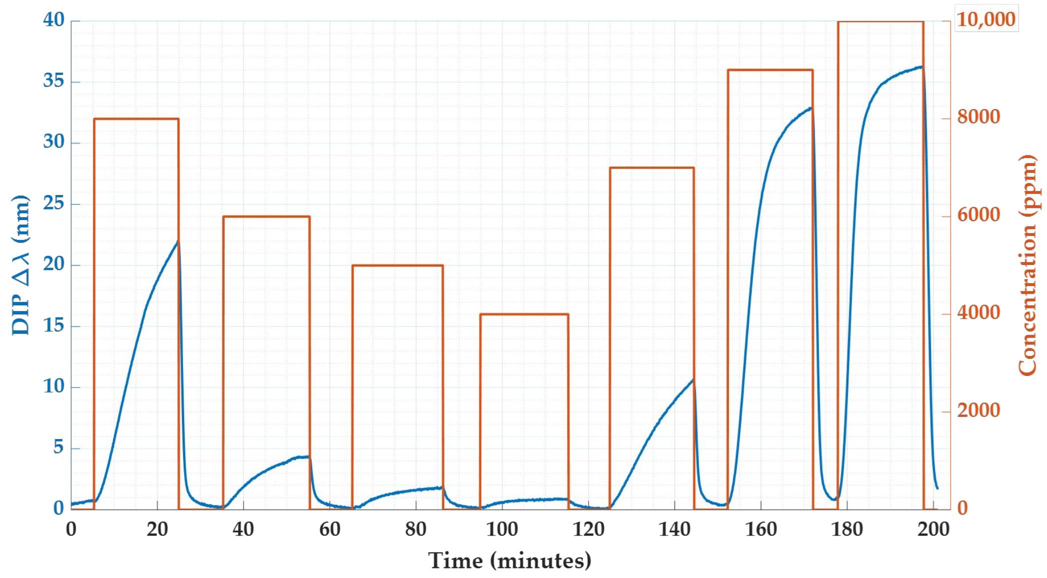

Figure S1.2 Gas sensing approach II. Red shift of the FP sensor (M3 minimum) with a 1550 nm ZIF-8 film (Figure 3d) towards EtOH pulses of 8000 ppm, 6000 ppm, 5000 ppm, 4000 ppm, 7000 ppm, 9000 ppm, and 10,000 ppm, respectively. This graph indicates that no poisoning takes place, thus confirming the reversibility of the behaviour.

S2. Sigmoidal fitting of experimental spectral shifts under EtOH pulses for LoD estimation

The lowest concentration that has been tested so far with our current analog mass flow control system via approach II gas sensing experiments is 4000 ppm. Figure S1.2 (or Figure 3b) and Figure S4.3 (or Figure 6b) for FP and SPR-G samples, respectively, show that there is still space to detect below this concentration. In order to roughly estimate the LoD of the current sensor, the M3 wavelength (for FP samples and 20 minutes after the start of the EtOH pulse) and the resonance dip wavelength (for SPR-G samples and 30 minutes after the start of the EtOH pulse) have been plotted vs. the EtOH concentration. The data have been adjusted with a sigmoidal fit, which shows that the LoD is around 3000 ppm, although further experiments must be performed to accurately determine the sensor limit.

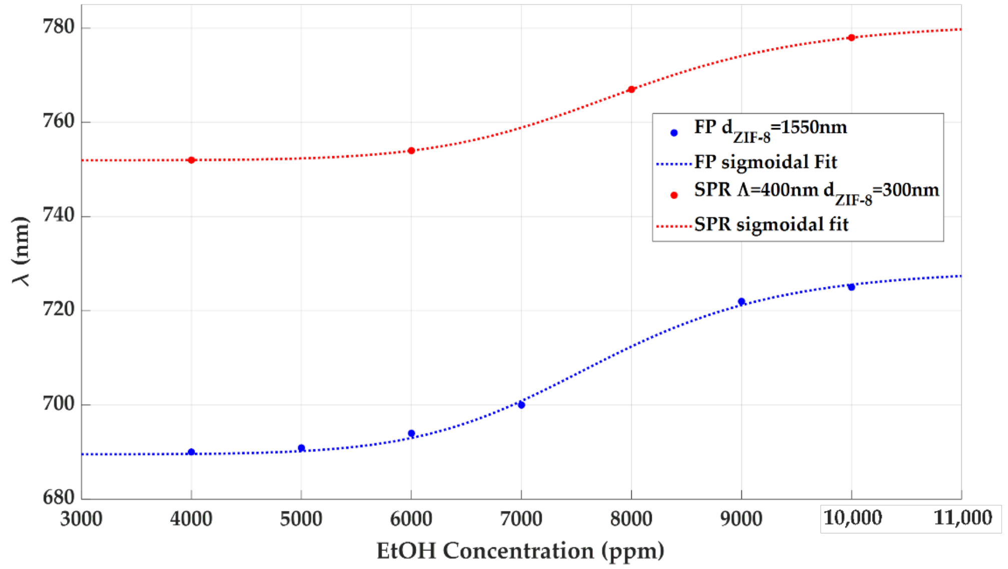

Figure S2.1 M3 wavelength (for FP sample with 1550 nm of ZIF-8 and 20 minutes after the introduction of the EtOH, in blue) and resonance dip wavelength (for SPR-G sample in the Λ=400 nm with 300 nm of ZIF-8 and 30 minutes after the introduction of the EtOH, in red) in front of EtOH concentrations. A sigmoidal fit has been used to adjust the data and roughly estimate the LoD of the sensors, which can be found around 3000 ppm.

S3. Fabry–Pérot interferometry to obtain RI of ZIF-8 film

In FP samples, the ZIF-8 film thickness is represented as the cavity length in Fabry–Pérot interferometry. In this manner, simulations have been made to adjust the experimental curves under N_2 and EtOH atmospheres for the 1550 nm ZIF-8 layer (curves of Figure 3b). To do so, Fabry–Pérot interferometry theory has been used [48] and is explained below.

The reflectivity comes from the reflected light in the air/ZIF-8 interface and that reflected in the ZIF-8/silicon substrate. The measured reflectivity is the result of the interference of these two waves and can be analysed by the following expression:

$$I_t = I_1 + I_2 + 2\sqrt{I_1 \cdot I_2} cos\left(\frac{4\pi nL}{\lambda}\right)$$

where L is the ZIF-8 thickness and n is the refractive index. The minimum values (minimums of the curves) of the expression are obtained as

$$cos\left(\frac{4\pi nL}{\lambda}\right) = -1 \Rightarrow \frac{4\pi nL}{\lambda_m} = (2m+1)\pi \qquad m = integer$$

Taking this into account and the considering the wavelength values at the experimental minimums, then $n_{ZIF-8,N_2} \cdot L = 1962$ and $n_{ZIF-8,EtOH} \cdot L = 2140$ for N_2 and EtOH, respectively. Fixing the thickness to 1550 nm, obtained with the profilometer, the refractive index values obtained are $n_{ZIF-8,N_2} = 1.26$ and $n_{ZIF-8,EtOH} = 1.38$. However, the interference patterns are better fit with values $n_{ZIF-8,N_2} = 1.24$ and $n_{ZIF-8,EtOH} = 1.35$ (Figure S3.1). These differences can be due to discrepancies in the real thickness at the sensing point and a dependence of the refractive index

on the wavelength. Nonetheless, all values are in accordance with other reported ZIF-8 refractive indexes [36,47,60-63].

In Figure S.3, the upper dashed lines belong to the experimental data (Figure 3b), while the lower dotted lines are the previous ones multiplied by the typical silicon reflectivity. The solid lines are obtained by applying the analytical expression with the proposed RI for N_2 and EtOH.

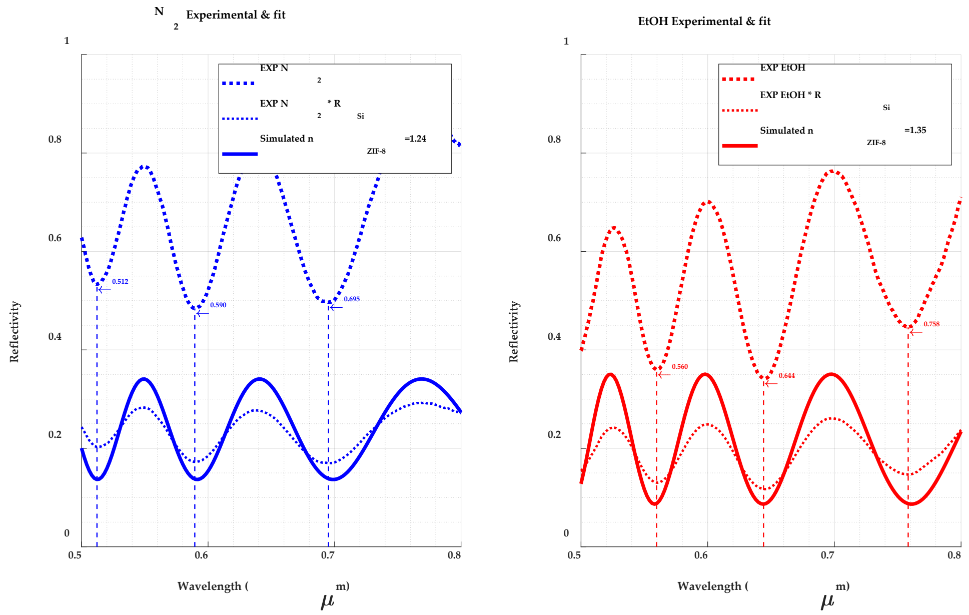

Figure S3.1 Experimental curves (dashed lines), experimental curves multiplied by the typical silicon reflectivity (dotted lines), and simulated curves (solid lines) for FP sample with 1550 nm of ZIF-8 under a N_2 atmosphere (left) and saturated EtOH (right). As can be seen, the interference patterns are better fit with values $n_{ZIF-8,N_2} = 1.24$ and $n_{ZIF-8,EtOH} = 1.35$.

S4. Gas sensing measurements in SPR-G samples

Gas sensing measurements have been performed on SPR-G samples with Λ = 400 nm and Λ = 500 nm DGs covered with ZIF-8 films via two different approaches. In approach I (Figure S4.1 and Figure S4.2), 1 µl of different EtOH/water solutions was manually added into the chamber with a micropipette without contacting the sample and was naturally allowed to evaporate. N_2 flow was then used to clean the chamber in between concentrations. In approach II (Figure S4.3), different EtOH concentrations were introduced in gas form into the chamber (concentrations were adjusted by controlling N_2 and EtOH flows from the bottles and maintaining the flow rate at 200 sccm). N_2 flow was also used to clean the chamber in between pulses of EtOH.

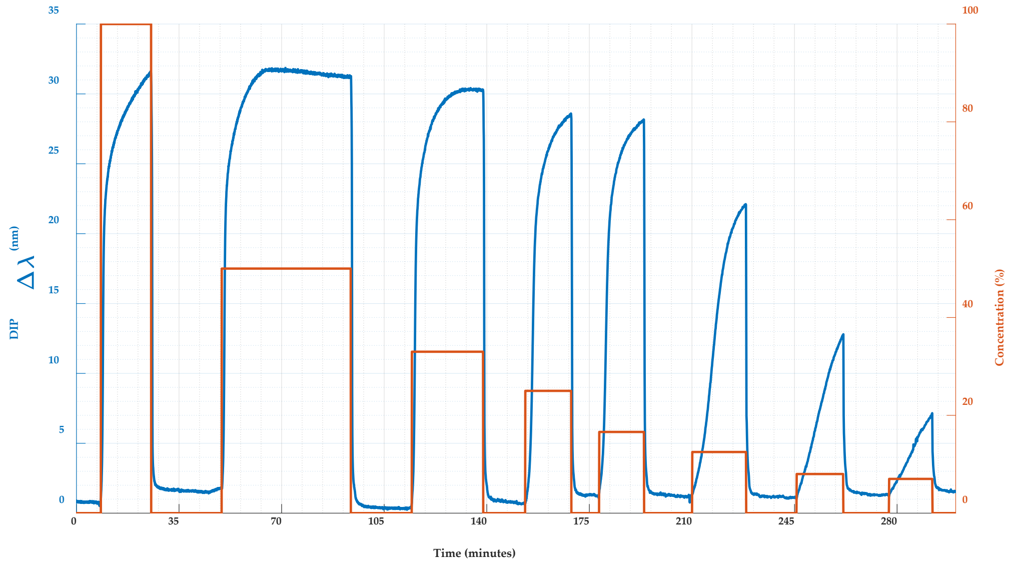

Figure S4.1 Gas sensing approach I. Red shift of the Λ = 400 nm DG covered with 300 nm of ZIF-8 towards EtOH pulses of 100%, 50%, 33%, 16%, 12%, 8%, and 7%, respectively.

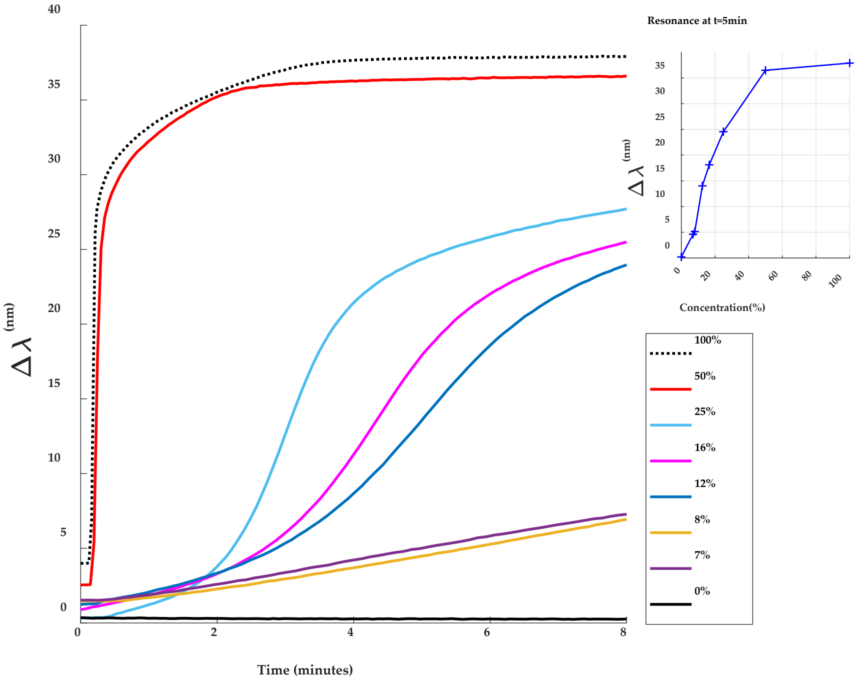

Figure S4.2 Gas sensing approach I. Red shift of the Λ = 500 nm DG covered with 300 nm of ZIF-8 towards EtOH pulses of 100%, 50%, 25%, 16%, 12%, 8%, and 7%, respectively.

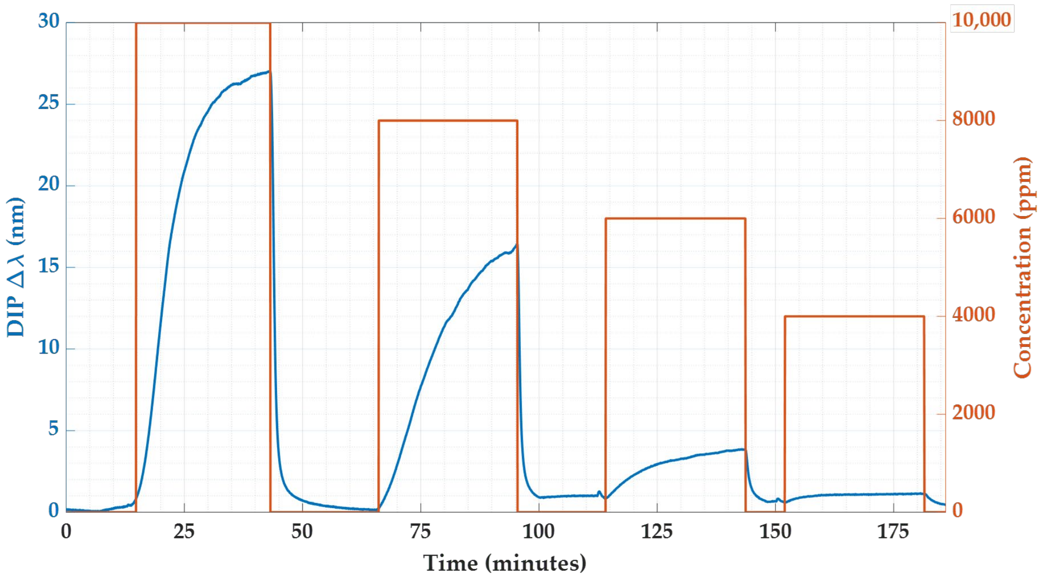

Figure S4.3 Gas sensing approach II. Red shift of the Λ = 500 nm DG covered with 300 nm of ZIF-8 towards EtOH pulses of 10,000 ppm, 8000 ppm, 6000 ppm, and 4000 ppm, respectively.
